# Apoptosis inhibitor of macrophage (AIM)/CD5L is involved in the pathogenesis of COPD

**DOI:** 10.1186/s12931-023-02508-0

**Published:** 2023-08-17

**Authors:** Michiko Takimoto-Sato, Masaru Suzuki, Hiroki Kimura, Haiyan Ge, Munehiro Matsumoto, Hironi Makita, Satoko Arai, Toru Miyazaki, Masaharu Nishimura, Satoshi Konno

**Affiliations:** 1 Department of Respiratory Medicine, Faculty of Medicine, Graduate School of Medicine, Hokkaido University, North 15 West 7, Kita-ku, Sapporo, 060-8638 Japan; 2 Division of Pulmonary, Critical Care and Sleep Medicine, Department of Medicine, Icahn School of Medicine at Mount Sinai, New York, United States of America; 3 Department of Respiratory and Critical Care Medicine, Huadong Hospital, Fudan University, Shanghai, China; 4 Hokkaido Medical Research Institute of Respiratory Diseases, Sapporo, Japan; 5 Laboratory of Molecular Biomedicine for Pathogenesis, Center for Disease Biology and Integrative Medicine, Faculty of Medicine, University of Tokyo, Tokyo, Japan; 6 The Institute for AIM Medicine, Tokyo, Japan; 7 LEAP, Japan Agency for Medical Research and Development, Tokyo, Japan

**Keywords:** Chronic obstructive lung disease, Apoptosis inhibitor of macrophage, Matrix metalloprotease-12, Alveolar macrophage

## Abstract

**Background:**

Alveolar macrophages (AMs) and AM-produced matrix metalloprotease (MMP)-12 are known to play critical roles in the pathogenesis of chronic obstructive pulmonary disease (COPD). The apoptosis inhibitor of the macrophages (AIM)/CD5 molecule-like (CD5L) is a multifunctional protein secreted by the macrophages that mainly exists in the blood in a combined form with the immunoglobulin (Ig)M pentamer. Although AIM has both facilitative and suppressive roles in various diseases, its role in COPD remains unclear.

**Methods:**

We investigated the role of AIM in COPD pathogenesis using porcine pancreas elastase (PPE)-induced and cigarette smoke-induced emphysema mouse models and an in vitro model using AMs. We also analyzed the differences in the blood AIM/IgM ratio among nonsmokers, healthy smokers, and patients with COPD and investigated the association between the blood AIM/IgM ratio and COPD exacerbations and mortality in patients with COPD.

**Results:**

Emphysema formation, inflammation, and cell death in the lungs were attenuated in AIM^−/−^ mice compared with wild-type (WT) mice in both PPE- and cigarette smoke-induced emphysema models. The PPE-induced increase in MMP-12 was attenuated in AIM^−/−^ mice at both the mRNA and protein levels. According to in vitro experiments using AMs stimulated with cigarette smoke extract, the MMP-12 level was decreased in AIM^−/−^ mice compared with WT mice. This decrease was reversed by the addition of recombinant AIM. Furthermore, an analysis of clinical samples showed that patients with COPD had a higher blood AIM/IgM ratio than healthy smokers. Additionally, the blood AIM/IgM ratio was positively associated with disease severity in patients with COPD. A higher AIM/IgM ratio was also associated with a shorter time to the first COPD exacerbation and higher all-cause and respiratory mortality.

**Conclusions:**

AIM facilitates the development of COPD by upregulating MMP-12. Additionally, a higher blood AIM/IgM ratio was associated with poor prognosis in patients with COPD.

**Trial Registration:**

This clinical study, which included nonsmokers, healthy smokers, and smokers with COPD, was approved by the Ethics Committee of the Hokkaido University Hospital (012–0075, date of registration: September 5, 2012). The Hokkaido COPD cohort study was approved by the Ethics Committee of the Hokkaido University School of Medicine (med02-001, date of registration: December 25, 2002).

**Supplementary Information:**

The online version contains supplementary material available at 10.1186/s12931-023-02508-0.

## Background

Chronic obstructive pulmonary disease (COPD) is primarily caused by cigarette smoke (CS) and characterized by airflow obstruction, emphysema, and airway inflammation. It is the third leading cause of death and poses a substantial social burden worldwide [1, 2]. As there is no drastic treatment for COPD that can significantly improve the prognosis, there is a need to understand the pathogenic mechanisms of the disease in detail. The alveolar macrophages (AMs) and neutrophils play an important role in the destruction of the alveolar walls by releasing proteases, cytokines, and chemokines, resulting in airspace enlargement [3, 4]. In particular, the AMs release matrix metalloprotease (MMP)-12, which promotes emphysema [5]. However, the pathophysiological mechanisms involved have not been fully elucidated.

Apoptosis inhibitor of macrophage (AIM), also known as a CD5 molecule-like (CD5L) or Spα, is a member of the scavenger receptor cysteine-rich superfamily [6, 7]. It was initially identified as an apoptosis inhibitor [7]. Subsequently, several of its functions were discovered. AIM regulates lipid metabolism [8] and operates as a critical switch for Th17 cells to a pathogenic state [9]. It promotes recovery from acute kidney injury by enhancing intraluminal debris clearance [10] and ameliorates ischemic stroke by attenuating danger associated molecular patterns [11]. AIM is also involved in the pathogenesis of hepatocellular carcinoma [12–14]. Although AIM has both positive and negative effects on organisms, it has been shown to react in a facilitative manner in inflammatory conditions [15–18]. Most studies have reported its facilitative role in the pathogenesis of respiratory diseases. We previously reported that AIM promoted lung inflammation in a lipopolysaccharide-induced lung injury mouse model [19]. High serum AIM levels predict acute lung parenchymal injury or acute respiratory disease syndrome in patients with trauma [20], and AIM has been suggested as an extracellular vesicle-derived biomarker of lung cancer [21]. AIM contributes to the development of methicillin-resistant *Staphylococcus aureus*-induced pneumonia [22] and chronic *Mycobacterium avium* infection in the lungs [23]. In contrast, AIM suppresses allergic airway inflammation in asthma [24].

There are several reports on the association between AIM and COPD, suggesting a harmful role. AIM overexpression in the alveolar type II epithelial cells contributes to emphysema formation in a transgenic mice model [25]. AIM has been shown to exist in the AMs and maintain their survival in the lungs of patients with COPD [26]. In rats, AIM gene expression is elevated by welding fumes exposure [27]. Additionally, the gene expressions of CD36 and scavenger receptor-1, which are both positively related to AIM expression, were upregulated in the AMs of smokers [28]. Therefore, AIM may facilitate the pathogenesis of COPD. However, little is known about its detailed mechanism of action, including its association with MMP-12.

We hypothesized that AIM promotes the development of COPD. To address this, we assessed lung inflammation, emphysema formation, and cell death in both elastase- and CS-induced emphysema models using AIM^−/−^ mice. Levels of MMP-12, cytokines, and chemokines were also evaluated. Next, we conducted in vitro experiments using mouse AMs cultured with CS extract (CSE), some of which contained recombinant mouse AIM (rAIM). Furthermore, we measured and analyzed the differences in the blood AIM/immunoglobulin (Ig)M ratio among nonsmokers, healthy smokers, and patients with COPD.

## Methods

### PPE-induced emphysema mouse model

Male C57BL/6 (wild-type [WT]) mice were purchased from CLEA Japan (Tokyo, Japan). AIM^−/−^ mice were created as previously reported [7]. Seven-to-nine-week old mice were anesthetized through isoflurane inhalation and intratracheally administrated with 0.2 U PPE (elastase from porcine pancreas; Sigma Aldrich, St. Louis, MO, USA) diluted with phosphate-buffered saline (PBS) to 40 µL on days 0, 7, and 14, whereas control animals received only 40 µL PBS. The mice were euthanized on days 0, 1, 3, 7, 14, and 21. All animal experimental protocols were approved by the Institutional Animal Care and Use Committee of the Hokkaido University.

### CS exposure of the mice

In this study, 8-to-10-week old male C57BL/6 mice (WT and AIM^−/−^) were exposed to CS or air as previously described [29]. Briefly, the mice were exposed to CS through their noses for 60 min in the CS generation and exposure system SG-200 (Sibata Scientific Technology, Tokyo, Japan) for up to 16 weeks (once daily for 5 days per week). The CS was generated from the mainstream of fresh cigarettes (12 mg tar and 1.0 mg nicotine, Marlboro; Philip Morris, Richmond, VA, USA), which was diluted with compressed air to the concentration of 5%. The total particulate matter mass concentration of 5% CS was stable within 1,255 ± 26 mg/m^3^. The control group was exposed to the air. The mice were euthanized on days 3, 7, 14, and 28 (short-term exposure) and at 16 weeks (long-term exposure).

### Morphometric assessment

For morphometric assessment, the lungs were inflated and fixed intratracheally with 10% formalin (Mildform 10 N; FUJIFILM Wako Pure Chemical Corporation, Osaka, Japan) at a static pressure of 25 cm H_2_O for 5 min. Formalin-fixed paraffin-embedded blocks were prepared and stained with hematoxylin and eosin (H&E). One large image per mouse was created from an H&E-stained section using a microscope (BZ-9000; Keyence, Osaka, Japan). The images were added with regularly aligned parallel grid lines with a length of 100 μm, spacing of 100 μm, and distance of 200 μm using the Image-Pro Plus software ver. 7.0 (Media Cybernetics, Rockville, MD, USA). The longest-aligned group in the image was selected, and the number of intersections of the lines in the group and alveolar septa were counted. Lines that crossed any vessels or bronchi were excluded. The mean linear intercept (MLI) was calculated by dividing the total length of the lines in the group by the number of intersections [30].

### Analysis of mouse bronchoalveolar lavage fluid (BALF)

The mice were intubated and subjected to bronchoalveolar lavage (BAL) with 0.6 mL normal saline three times through a tracheal cannula. Total cell counts in the BALF were calculated using a hemocytometer. Red blood cells were removed using the Red Blood Cell Lysing Buffer Hybri-Max (Sigma-Aldrich). Cytospun BALF was stained with Diff-Quik Reagent (Sysmex, Kobe, Japan), and differential cell counts were calculated by counting > 400 cells per mouse using a microscope. After performing the BAL, the lungs were inflated with the Tissue-Tek O.C.T. Compound (Sakura Finetek Japan, Tokyo, Japan) (50% vol/vol) diluted with PBS containing 10% sucrose and stored at -80 °C. Proteins in the BALF were assessed using enzyme-linked immunosorbent assay (ELISA): interleukin (IL)-33 (Mouse IL-33 DuoSet ELISA; R&D Systems, Minneapolis, CA, USA) and MMP-12 (Mouse MMP-12 Kit Price PicoKine; BOSTER Biological Technology, Pleasanton, CA, USA).

### Culture of mouse AMs with CSE and recombinant AIM protein

BALF was collected from male C57BL/6 mice (WT and AIM^−/−^) by washing the lungs with 1.0 mL saline 15 times. Subsequently, the cells in the BALF were incubated in RPMI 1640 with 10% fetal bovine serum and 100 U/mL penicillin/streptomycin in a 24-well plate for 1 h. After washing twice with PBS, the adherent cells were used as AMs [31]. CSE was prepared as previously described [31]. Briefly, the mainstream smoke of two cigarettes (Marlboro) was bubbled through 15 mL of culture media and filtered through a 0.22-µm filter (defined as 100% CSE). The CSE was diluted to a concentration of 1% using culture medium. Recombinant murine AIM protein was produced in-house as previously described [7]. The AMs were incubated with 1% CSE, and 1 µg/mL rAIM was added to those derived from AIM^−/−^ mice every 6 h. After incubation, the cells were harvested for RNA isolation at 6 and 12 h. The culture media were also collected, and MMP-12 was assessed using ELISA (Mouse MMP-12 Kit Price PicoKine; BOSTER Biological Technology).

### TdT-mediated dUTP nick end labeling (TUNEL) assay

Lung sections were stained using the TUNEL method using the In Situ Cell Death Detection Kit, Fluorescein (Roche, Indianapolis, IN, USA) to evaluate cell death. The number of TUNEL-positive cells was counted under a microscope, and 12 random fields were assessed per mouse.

### RNA purification and quantitative real-time polymerase chain reaction (PCR)

Total RNA was extracted using the RNeasy Mini Kit (Qiagen, Hilden, Germany). RNA was reverse transcribed using a High-Capacity RNA-to-cDNA Kit (Applied Biosystems, Foster City, CA, USA) on the GeneAmp PCR System 9700 (Applied Biosystems). TaqMan Gene Expression Assays probes (Applied Biosystems) were used for mice MMP-12 (Mm00500554_m1), MMP-9 (Mm00442991_m1), IL-1β (Mm00434228_m1), IL-5 (Mm00439646_m1), IL-6 (Mm00446190_m1), IL-33 (Mm0050403_m1), tumor-necrosis factor (TNF)-α (Mm00443258_m1), interferon (IFN)-γ (Mm01168134_m1), C-C motif chemokine 2 (CCL2) (Mm00441242_m1), and β_2_-microglobulin (Mm00437764_m1). Quantitative reverse transcription (qRT)-PCR was performed using the StepOnePlus Real-Time PCR System (Applied Biosystems). The relative standard curve method was used to quantify the mRNA levels. All values were expressed as ratios to mouse β_2_-microglobulin, which was used as the internal control.

### Measurement of human plasma AIM and IgM levels in nonsmokers, healthy smokers, and smokers with COPD

We recruited nonsmokers, healthy smokers (without COPD), and smokers with COPD at the Hokkaido University Hospital (Sapporo, Japan) and collected peripheral blood samples from March to May 2012. All participants were aged 40 years or above. Nonsmokers, healthy smokers, and smokers with COPD were defined as having smoking histories of ≤ 10 pack-years (also being smoke-free for more than 10 years at the time of recruitment), ≥ 20 pack-years, and ≥ 40 pack-years, respectively. Patients with COPD were defined as having emphysema in the chest computed tomography scan and showed < 70% postbronchodilator forced expiratory volume in 1 s/forced vital capacity. Emphysema was visually assessed by pulmonologists according to the modified Goddard scoring system as previously described [32]. The severity of emphysema on CT was scored by the percentage of low-attenuation area in the entire lung field and < 5% affected was defined as “no emphysema”. Patient information, including age, sex, and pulmonary function, was collected from medical records. The exclusion criteria were as follows: patients who had other chronic respiratory diseases, including asthma or chronic lower airway infections, and hematologic malignancies; who were undergoing cytotoxic anticancer treatment for malignant tumors; who had neutrophil dysfunction; who had experienced COPD exacerbations or lung infections within 1 month; and who were defined ineligible by a principal investigator. Fifty participants (16 nonsmokers, 15 healthy smokers, and 19 smokers with COPD) were analyzed in this study. This study was approved by the Ethics Committee of the Hokkaido University Hospital (012–0075) and conducted in accordance with the Declaration of Helsinki. Written informed consent was obtained from all participants. We evaluated the AIM-to-IgM ratio to reduce the impact of fluctuating IgM levels, as AIM is known to exist mainly in the IgM-binding form in the blood, and its level correlates with the IgM levels [33]. Plasma levels of AIM and IgM were assessed using ELISA: AIM (Human Apoptosis Inhibitor of Macrophage ELISA kit; Trans Genic Inc, Kobe, Japan) and IgM (Human IgM ELISA Quantitation Set; Bethyl Laboratories, Montgomery, TX, USA). Associations between the plasma AIM/IgM ratios among the three groups were analyzed.

### Measurement of human serum AIM and IgM levels in patients with COPD

Patients with COPD were recruited for the Hokkaido COPD cohort study from the Hokkaido University Hospital and its nine affiliated hospitals from May 2003 to April 2005, as previously described [32, 34–38]. The study protocol was approved by the Ethics Committee of Hokkaido University School of Medicine (med02-001) and written informed consent was obtained from all participants. Of the 300 patients with COPD who were initiated for follow-up, 21 patients who met the exclusion criteria and 152 patients who could not be measured due to insufficient samples were excluded. Finally, 133 patients with COPD with available data were analyzed. Serum AIM and IgM levels were assessed using the above-mentioned kits. The associations of the serum AIM/IgM ratio with the degree of airflow limitation at baseline, development of the first COPD exacerbation requiring a prescription change for 5 years, and all-cause and respiratory mortality for 10 years were analyzed.

### Statistical analysis

Data from animal experiments were presented as mean ± SEM. All animal experiments were performed at least twice except for CS exposure to mice. Differences between the two groups were analyzed using the Welch’s t*-*test (with the Holm’s adjustment for multiple evaluations) or Mann–Whitney U test. One-way analysis of variance was used to compare differences between more than two groups, followed by the post hoc Tukey–Kramer or Dunnett’s test. In clinical research, data were expressed as mean ± SEM, mean ± standard deviation, or number (%). Differences between the groups were examined using the Kruskal–Wallis, chi-square, and Tukey–Kramer tests. Trends among the groups were analyzed using the Jonckheere–Terpstra test. Survival rates were analyzed using the Kaplan–Meier method with the log-rank trend test and the multivariate Cox proportional hazards model. Serum AIM/IgM ratio, age, and current smoking status at the recruitment were included as covariates. All statistical analyses were performed using the GraphPad Prism ver. 8.4.3 (GraphPad Software, San Diego, CA, USA) or EZR ver.1.61 (Saitama Medical Center, Jichi Medical University, Saitama, Japan), a graphical user interface for the R software (The R Foundation for Statistical Computing, Vienna, Austria) [39]. Statistical significance was defined as p < 0.05.

## Results

### Lung inflammation, emphysema formation, cell death, and MMP-12 levels were attenuated in the AIM^−/−^mice in the PPE-induced emphysema model

**Fig. 1:**
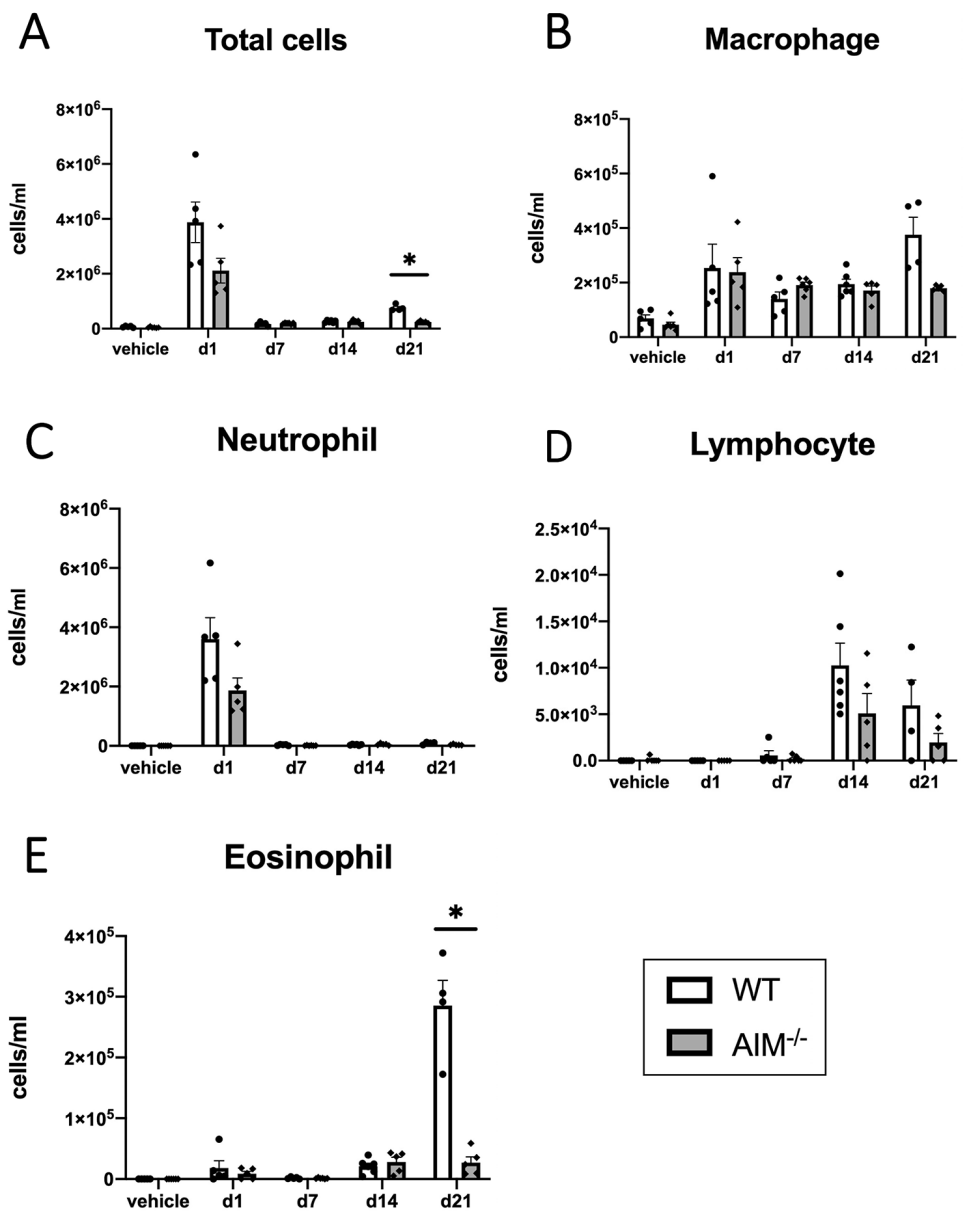
Lung inflammation assessed BALF from PPE-treated mice. (**A**) The number of total cells, (**B**) the number of macrophages, (**C**) the number of neutrophils, (**D**) the number of lymphocytes, and (**E**) the number of eosinophils in the BALF. Data are expressed as mean ± SEM (n = 4–6). ^*^p < 0.05 by the Welch’s t test

We first assessed the number of inflammatory cells in the BALF collected from PPE-treated mice at different time points. On day 21, the numbers of total inflammatory cells and eosinophils were significantly decreased in the AIM^−/−^ mice compared with the WT mice (Fig. 1A, E). Similar tendencies were observed in the macrophages, neutrophils, and lymphocytes; however, the differences were not statistically significant (Fig. 1B–D).

**Fig. 2:**
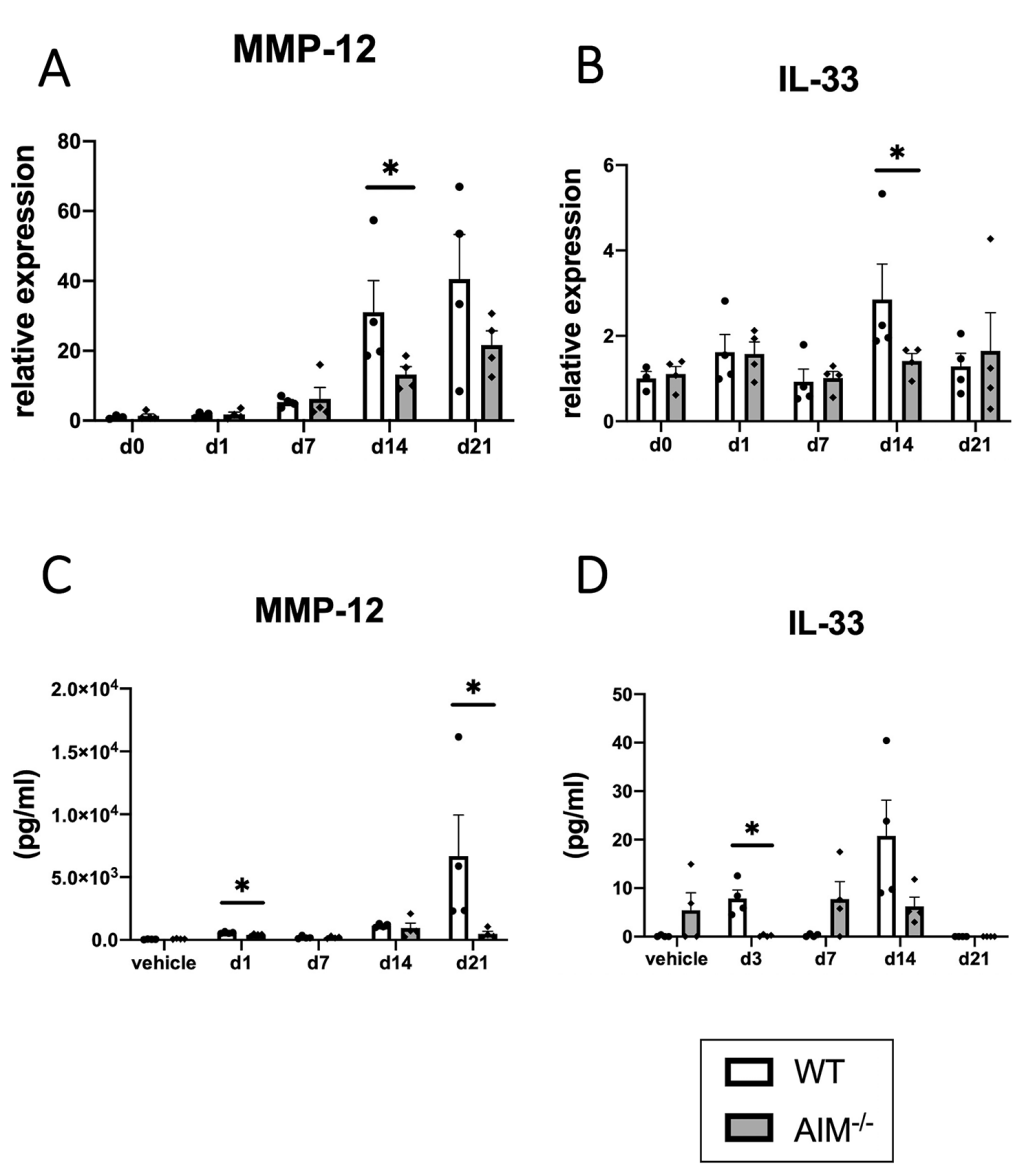
Gene expressions and protein levels in the lungs from PPE-treated mice. Gene expression in the lungs and protein levels in the BALF during PPE treatment were assessed using qRT-PCR or ELISA. (**A**) MMP-12 mRNA, (**B**) IL-33 mRNA, (**C**) MMP-12 protein, and (**D**) IL-33 protein. Data are expressed as mean ± SEM (n = 3–4). ^*^p < 0.05 by the Mann–Whitney U test

Next, we evaluated the gene expressions of cytokines, chemokines, and MMPs in the whole lungs of PPE-treated mice using qRT-PCR. The AIM^−/−^ mice showed significantly lower MMP-12 and IL-33 gene expression levels 14 days after PPE treatment than the WT mice (Fig. 2A, B). Although IL-5 showed a similar tendency, there were no differences in the gene expression of MMP-9, other cytokines, or chemokines (Additional file 1, Figure S1A–G). MMP-12 and IL-33 protein levels in the BALF were also significantly suppressed in the AIM^−/−^ mice compared with those in the WT mice (Fig. 2C, D).

**Fig. 3:**
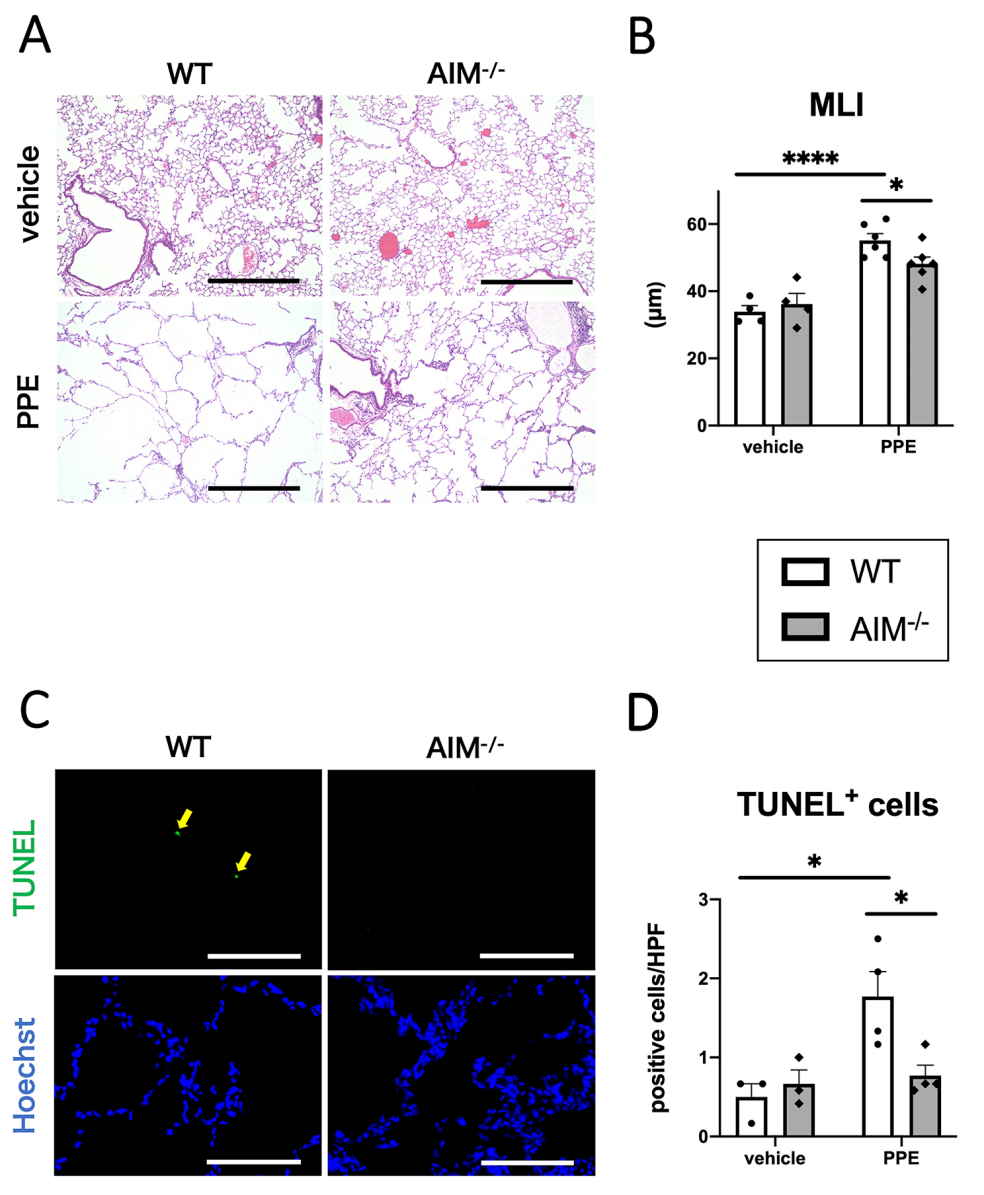
Emphysema formation and cell death in PPE-treated mice. (**A**) Representative photomicrographs of lungs of the AIM^−/−^ or WT mice on day 21 after PPE or vehicle treatment. Scale bar: 500 μm. (**B**) Quantification of MLI on day 21 after PPE or vehicle treatment. (**C**) Representative photomicrographs of TUNEL and Hoechst staining of lungs of the AIM^−/−^ or WT mice on day 21 after PPE or vehicle treatment. Scale bar: 100 μm. (**D**) Quantification of TUNEL-positive cells on day 21 after PPE or vehicle treatment. Data are expressed as mean ± SEM (n = 3–6). ^*^p < 0.05, ^****^p < 0.001 by the Welch’s t test with a Holm adjustment for (**B**) and the Tukey–Kramer test for (**D**)

To assess emphysema formation in the lungs on day 21, the MLI values were calculated using H&E-stained slides. In the WT mice, MLI was increased by PPE treatment, reflecting emphysema formation. In the PPE group, the MLI value was significantly lower in the AIM^−/−^ mice than in the WT mice (Fig. 3A, B). We performed TUNEL staining to assess cell death in the lungs of PPE-treated mice. In the WT mice, the number of TUNEL-positive cells was significantly increased by PPE treatment on day 21. The AIM^−/−^ mice had significantly fewer positive cells in the PPE group than the WT mice (Fig. 3C, D).

### Lung inflammation, emphysema formation, and cell death were attenuated in the AIM^−/−^ mice in the CS model

**Fig. 4:**
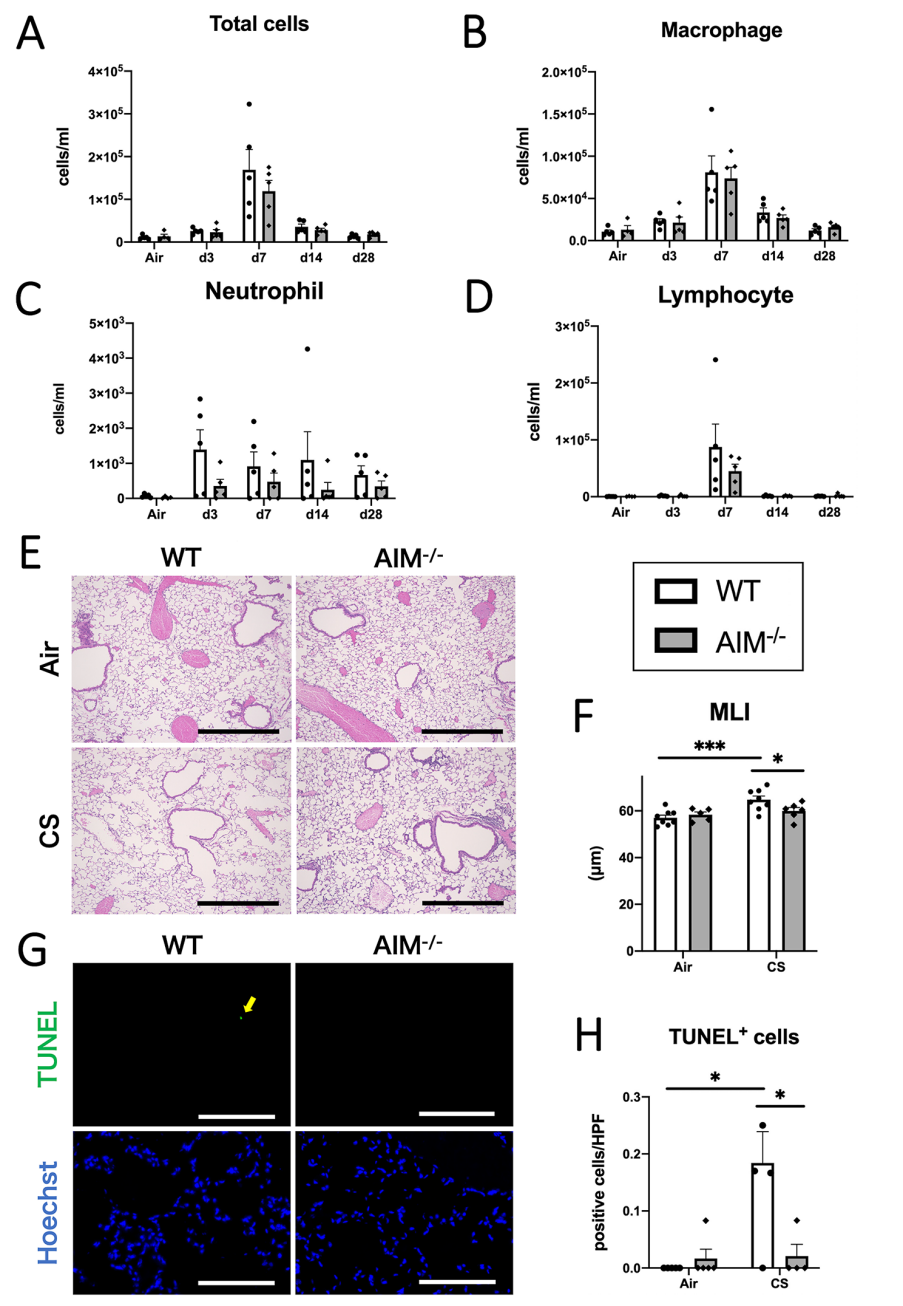
Lung inflammation, emphysema formation, and cell death in CS-exposed mice. (**A**) The numbers of total cells, (**B**) number of macrophages, (**C**) number of neutrophils, and (**D**) number of lymphocytes in the BALF. (**E**) Representative photomicrographs of lungs of the AIM^−/−^ or WT mice 16 weeks after CS or air exposure. Scale bar: 500 μm. (**F**) Quantification of MLI 16 weeks after CS or air exposure. (**G**) Representative photomicrographs of TUNEL and Hoechst staining of lungs of the AIM^−/−^ or WT mice after 16 weeks of CS or air exposure. Scale bar: 100 μm. (**H**) Quantification of TUNEL-positive cells 16 weeks after CS or air exposure. Data are expressed as mean ± SEM (n = 4–8). ^*^p < 0.05, ^**^p < 0.01, ^***^p < 0.005 by the Welch’s t test for (A)–(D), Welch’s t test with a Holm adjustment for (**F**), and Tukey–Kramer test for (**H**)

To confirm the findings observed in the PPE model, the mouse model of CS-induced emphysema was used next. The number of cells in the BALF from CS-exposed mice was quantified at different time points until day 28. The number of total inflammatory cells, lymphocytes, and neutrophils tended to be more suppressed in the AIM^−/−^ mice than in the WT mice (Fig. 4A, C, and D), although the differences were not statistically significant. There were no differences in macrophage counts (Fig. 4B). No eosinophils were detected at any time point.

We also measured the MLI to evaluate the degree of emphysema in the lungs at 16 weeks in the CS mouse model. In the WT mice, the MLI was significantly elevated following CS exposure. The MLI was significantly suppressed in the AIM^−/−^ mice in the CS group compared to that in the WT mice (Fig. 4E, F). Cell death in the lungs of CS-exposed mice was evaluated using TUNEL staining at 16 weeks. The number of TUNEL-positive cells significantly increased following CS exposure in the WT mice. In the CS group, the number of positive cells significantly decreased in the AIM^−/−^ mice than in the WT mice (Fig. 4G, H).

### In CSE-stimulated AMs from the AIM^−/−^ mice, MMP-12 increase was attenuated and recovered by the addition of rAIM

**Fig. 5:**
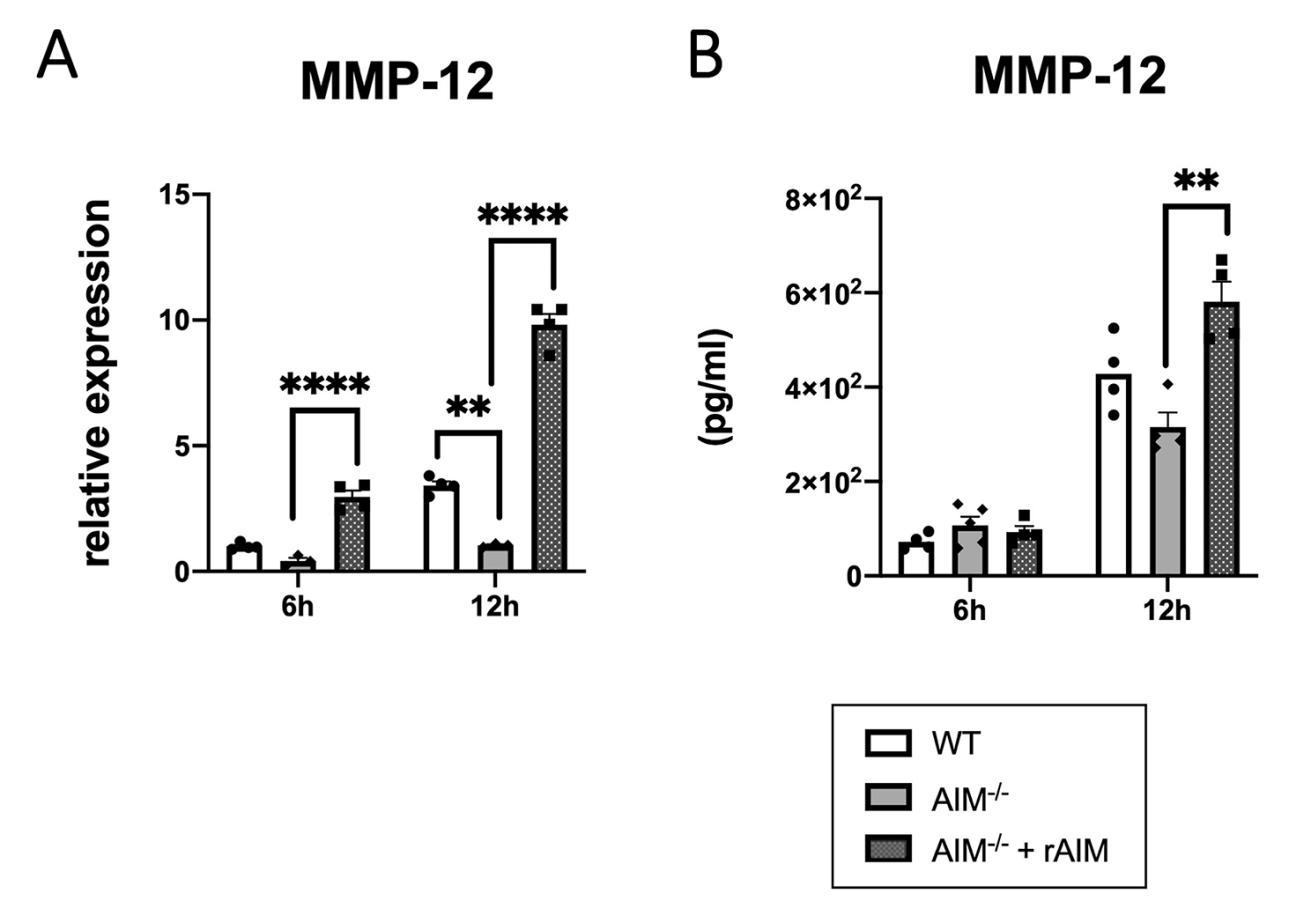
MMP-12 levels in the CSE-stimulated AMs. MMP-12 mRNA (**A**) and MMP-12 protein (**B**) levels in the CSE-stimulated AMs from the WT and AIM^−/−^ mice and CSE-stimulated AMs from AIM^−/−^ mice cultured with rAIM. Data are expressed as mean ± SEM (n = 3–5). ^**^p < 0.01, ^****^p < 0.001 by the Dunnett test

Because the AMs have been reported to be the main source of both AIM and MMP-12 in the lungs, we conducted an in vitro experiment using the AMs. The AMs derived from the WT or AIM^−/−^ mice were stimulated and incubated with 1% CSE, and MMP-12 levels were assessed using qRT-PCR and ELISA. MMP-12 gene expression was suppressed in the AIM^−/−^ mice compared with that in the WT mice, and the decrease in the AIM^−/−^ mice was reversed by the addition of rAIM (Fig. 5A). Similar results were observed for MMP-12 protein levels in the medium. In the AMs from the AIM^−/−^ mice, MMP-12 levels in the medium tended to be suppressed compared with those in the WT mice, whereas the decrease was recovered by rAIM addition at 12 h (Fig. 5B).

### Higher AIM/IgM ratio in the blood is associated with COPD and its poor prognosis

**Fig. 6:**
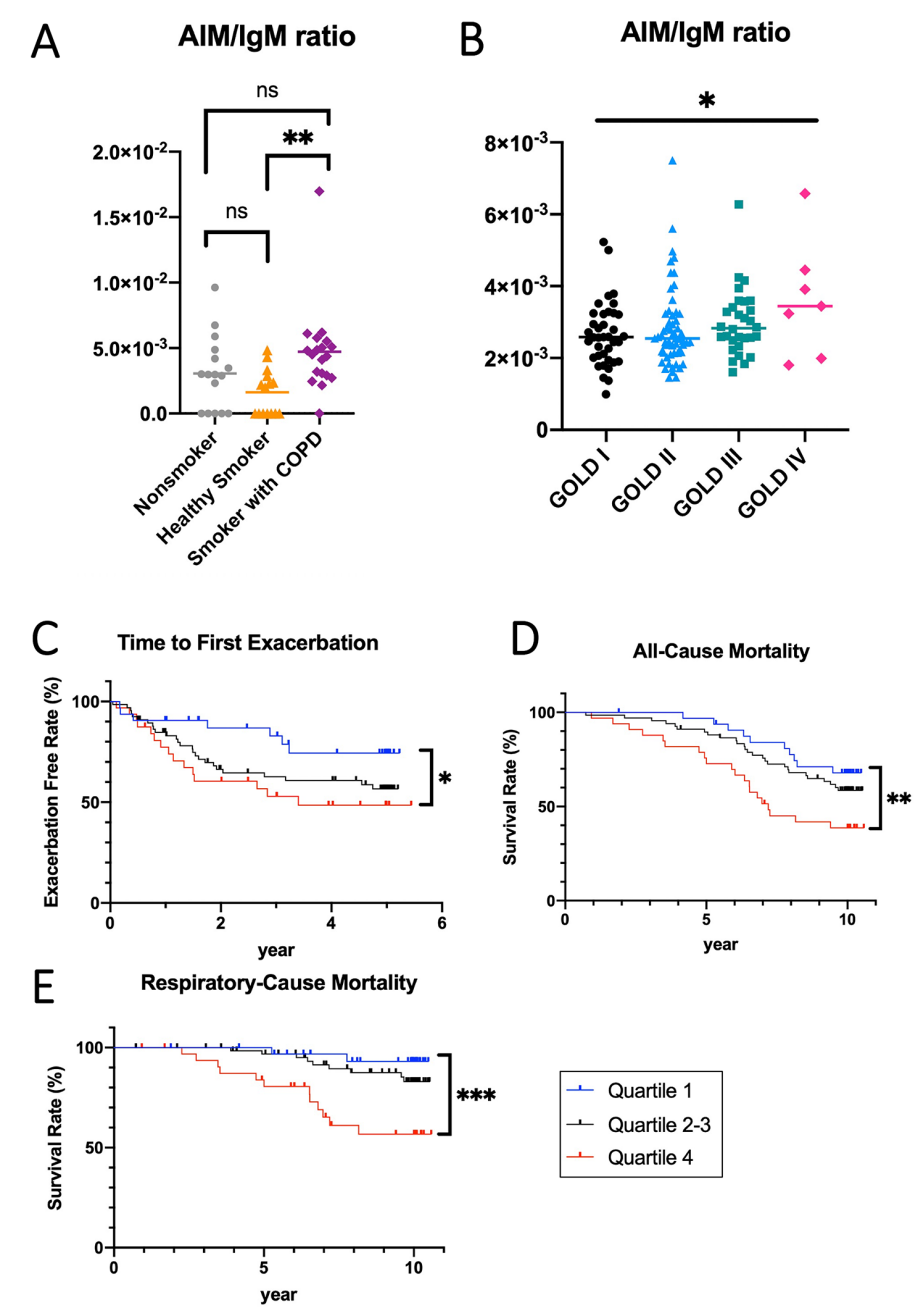
Blood AIM/IgM ratios in humans. (**A**) Plasma AIM/IgM ratio in nonsmokers, healthy smokers, and smokers with COPD. (**B**) Serum AIM/IgM ratio in patients with COPD classified by the degree of airflow limitation (GOLD classification). The Kaplan–Meier curves for time to first exacerbation (**C**), all-cause mortality (**D**), and respiratory-cause mortality (**E**) among patients with COPD classified by serum AIM/IgM ratio. Data are expressed as mean ± SEM. ^*^p < 0.05, ^**^p < 0.01, ^***^p < 0.005 by the Tukey–Kramer test for (**A**), Jonckheere–Terpsta test for (**B**), and log-rank trend test for (**C**)–(**E**).

Next, we assessed the blood AIM/IgM ratio in human samples. The plasma AIM/IgM ratios of the three patient groups (16 nonsmokers, 15 healthy smokers, and 19 smokers with COPD) were analyzed. There were no significant differences in age and sex among the three groups (Additional file 1, Table S1). Although we first intended to recruit healthy smokers with a lower smoking exposure history than patients with COPD as described in the recruitment criteria, there was no significant difference in pack-years between the 2 groups as a result. The plasma AIM/IgM ratio was significantly higher in smokers with COPD than in healthy smokers, whereas there were no differences between smokers with COPD and nonsmokers or between healthy smokers and nonsmokers (Fig. 6A).

We next evaluated the serum AIM/IgM ratio in the Hokkaido COPD cohort. The baseline characteristics of the patients are shown in Additional file 1, Table S2. There was a significant correlation between the GOLD severity and serum AIM/IgM ratio (Fig. 6B, GOLD 1 [n = 38], GOLD 2 [n = 58], GOLD 3 [n = 30], and GOLD 4 [n = 7]), although the values had considerable overlap among the 4 groups. We also assessed the prognosis of patients who were divided into quartiles according to their serum AIM/IgM ratios (quartile 1 [n = 33], quartile 2–3 [n = 67], and quartile 4 [n = 33]). Patients with higher serum AIM/IgM ratios had a shorter time to the first exacerbation, higher all-cause mortality, and higher respiratory-cause mortality (Fig. 6C–E). Multivariate Cox proportional hazards models revealed that 1 increase in the natural logarithm of serum AIM/IgM ratio was independently associated with higher all-cause and respiratory-cause mortality. It also tended to associate with a shorter time to the first exacerbation, although it did not reach a significant difference (Additional File 1, Table E3). Similarly, 1 older age was associated with all-cause and respiratory-cause mortality, although not with a time to the first exacerbation. Current smoking was not associated with the prognosis.

## Discussion

In the present study, we demonstrated that lung inflammation and airspace enlargement were attenuated in the AIM^−/−^ mice in both PPE- and CS-induced emphysema models. Cell death was also suppressed in the AIM^−/−^ mice. Additionally, the AIM^−/−^ mice showed decreased MMP-12 upregulation at both protein and mRNA levels in the PPE-induced emphysema model. In in vitro experiments using CSE-stimulated AMs, MMP-12 levels were also decreased in the AMs obtained from the AIM^−/−^ mice, but they recovered after the addition of rAIM.

MMP-12, also known as macrophage elastase, is a well-known factor that contributes to the development of COPD. In previous studies, MMP-12 levels in sputum were elevated in patients with COPD compared to those in nonsmokers or healthy smokers [40], and the MMP-12 gene was highly expressed in the AMs from smokers [41]. In animal studies, MMP-12 was essential for emphysema formation in a CS-induced mouse model [5] and for macrophage chemotactic activity in smoke-exposed lungs [42]. Furthermore, MMP-12 inhibitors reduce emphysema formation and lung inflammation [43–45]. The results of this study suggest that AIM facilitates COPD development via MMP-12 upregulation in mouse models. Increased MMP-12 production occurs through a variety of pathways [46]. CS-induced MMP-12 increase in the macrophages has been indicated to be promoted through proteinase-activated receptor-1(PAR-1) [43], granulocyte macrophage colony-stimulating factor (GM-CSF) [47], and TNF-α [48]. In our PPE model, TNF-α gene expression in the lungs was slightly suppressed in the AIM^−/−^ mice, which did not reach statistical significance.

In the present study, the increase of IL-33 was suppressed in the AIM^−/−^ mice in the PPE-induced model. However, the role of IL-33 in the development of emphysema remains controversial. Most previous studies have indicated its promoting role via eosinophilic inflammation [49–51]. However, a recent study reported that a complete loss of IL-33 enhances emphysema formation in both PPE- and CSE-induced models [52]. Doyle et al. reported that eosinophil-derived IL-13 contributes to alveolar destruction via MMP-12 induction [53]. However, in the present study, the IL-13 gene was barely expressed in the PPE model. If MMP-12 and eosinophilic inflammation are related in our model, a different pathway may exist. Furthermore, the PPE-induced increase of eosinophilic inflammation was attenuated in the AIM^−/−^ mice. Additionally, an increase in IL-5 gene expression in the lungs tended to be suppressed in the AIM^−/−^ mice, although almost no IL-4 or IL-13 gene expression was detected. There are several reports that mainly suggest a facilitative function of AIM in eosinophilic airway inflammation or asthma [54, 55]. It was also reported that an increase in AIM levels may induce eosinophilia in patients with atopic dermatitis [56]. In contrast, AIM suppresses allergic asthma by expanding CD11c^high^ AMs in mice [24]. The results of the present study suggest that AIM is also partially associated with IL-33-induced upregulation of type-2 inflammation, which leads to emphysema in the PPE model. Further studies are required to elucidate the mechanism by which AIM affects MMP-12 expression during the pathophysiology of COPD.

Cell death also contributes to the pathogenesis of COPD. Apoptosis increases more in the lungs of patients with COPD than in those of healthy controls [57]. In animal experiments, apoptosis was increased in CS-exposed emphysema mice [58]. In addition, smoking-induced macrophage and neutrophilic inflammation cause apoptosis in the lungs of patients with COPD [59]. In the present study, the AIM^−/−^ mice exhibited reduced number of airway inflammatory cells in both PPE and CS models. Furthermore, cell death was suppressed in the AIM^−/−^ mice compared to that in the WT mice. Kojima et al. reported that AIM exists in the AMs and inhibits the apoptosis of CSE-stimulated AMs by enhancing B-cell lymphoma-extra large (Bcl-xL) gene expression, although Bcl-xL plays only a partial role in the mechanism [26]. This indicates that by inhibiting the apoptosis of inflammatory cells, including macrophages, AIM may accelerate the death of cells comprising the airway walls, which reflects the destruction of lung structures and leads to emphysema formation.

We showed that patients with COPD had higher blood AIM/IgM ratios than healthy smokers. However, there were no significant differences between nonsmokers and patients with COPD or nonsmokers and healthy smokers. In the Hokkaido COPD cohort study, we demonstrated that the blood AIM/IgM ratio was positively associated with disease severity as assessed using the GOLD classification in patients with COPD. In addition, when patients were divided into three groups based on the blood AIM/IgM ratio, the time to first exacerbation was shorter in the group with a higher AIM/IgM ratio. All-cause and respiratory-cause mortality rates were lower in the high AIM/IgM ratio group. These results confirmed the facilitative role of AIM in the pathogenesis of COPD.

Our study had several limitations. First, because of unavailability of a large amount of rAIM, we were unable to confirm the recovery of emphysema and lung inflammation by administering rAIM to the AIM^−/−^ mice in the in vivo studies. However, we demonstrated that rAIM induced MMP-12 production in the AMs in vitro. Second, we focused only on the AMs but not on the interstitial macrophages (IMs). MMP-12 is believed to be produced predominantly by the AMs. However, a recent study proposed that IMs are the primary MMP-12 producers, and that the IL-4/IM/MMP-12 axis is important for the development of emphysema [60]. Although it was technically difficult to analyze the IMs in our study, further studies are required. Third, we were unable to determine the precise mechanism by which AIM promoted COPD via MMP-12. Further research is required to clarify this issue.

## Conclusions

We demonstrated that lung inflammation, emphysema formation, and cell death were attenuated in the AIM^−/−^ mice in both PPE- and CS-induced mouse models. The increase in MMP-12 protein and gene expression was suppressed in the AIM^−/−^ mice, both in the PPE model and CSE-stimulated AMs. We further showed that a high blood AIM/IgM ratio was associated with disease severity and poor prognosis in patients with COPD. These results suggest that AIM may facilitate the development of COPD, which requires MMP-12 upregulation.

### Electronic supplementary material

Below is the link to the electronic supplementary material.

**Additional File 1:** Table S1. Characteristics of the study patients with different smoking statuses. Table S2. Baseline characteristics in the Hokkaido COPD cohort. Table S3. Risk factors for prognosis in the Hokkaido COPD cohort.

## Data Availability

The authors declare that all data are available within the manuscript.

## List of abbreviations

AIM: apoptosis inhibitor of macrophage
CD5L: CD5 molecule-like
COPD: chronic obstructive pulmonary disease
AM: alveolar macrophage
MMP: matrix metalloprotease
Ig: immunoglobulin
PPE: porcine pancreas elastase
WT: wild-type
CS: cigarette smoke
CSE: cigarette smoke extract
rAIM: recombinant apoptosis inhibitor of macrophage
PBS: phosphate-buffered saline
H&E: hematoxylin and eosin
MLI: mean linear intercept
BALF: bronchoalveolar lavage fluid
BAL: bronchoalveolar lavage
ELISA: enzyme-linked immunesorbent assay
IL: interleukin
TUNEL: TdT-mediated dUTP nick end labeling
PCR: polymerase chain reaction
TNF: tumor necrosis factor
IFN: interferon
CCL2: C-C motif chemokine ligand 2
qRT-PCR: quantitative reverse transcription polymerase chain reaction
PAR-1: proteinase activated receptor-1
GM-CSF: granulocyte macrophage colony-stimulating factor
Bcl-xL: B-cell lymphoma-extra large
IM: interstitial macrophage
HR: hazard ratio
CI: confident interval
