# Supplementary material for: Apoptosis inhibitor of macrophage (AIM)/CD5L is involved in the pathogenesis of COPD
Source: Respir Res. 2023 Aug 17;24:201. doi: 10.1186/s12931-023-02508-0 (PMC10433671; doi:10.1186/s12931-023-02508-0)
Supplement: Supplementary file 1 — Additional File 1: Table S1. Characteristics of the study patients with different smoking statuses. Table S2. Baseline characteristics in the Hokkaido COPD cohort. Table S3. Risk factors for prognosis in the Hokkaido COPD cohort. [file 12931_2023_2508_MOESM1_ESM.docx]

Additional file 1

**Apoptosis inhibitor of macrophage (AIM)/CD5L is involved in the pathogenesis of COPD**

Michiko Takimoto-Sato^1^, Masaru Suzuki^1^, Hiroki Kimura^1,2^, Haiyan Ge^3^, Munehiro Matsumoto^1^, Hironi Makita^4^, Satoko Arai^5,6^, Toru Miyazaki^6,7^, Masaharu Nishimura^1,4^, Satoshi Konno^1^

1. Department of Respiratory Medicine, Faculty of Medicine and Graduate School of Medicine, Hokkaido University, Sapporo, Japan.
2. Division of Pulmonary, Critical Care and Sleep Medicine, Department of Medicine, Icahn School of Medicine at Mount Sinai, New York, United States of America.
3. Department of Respiratory and Critical Care Medicine, Huadong Hospital, Fudan University, Shanghai, China.
4. Hokkaido Medical Research Institute of Respiratory Diseases, Sapporo, Japan.
5. Laboratory of Molecular Biomedicine for Pathogenesis, Center for Disease Biology and Integrative Medicine, Faculty of Medicine, University of Tokyo, Japan.
6. The Institute for AIM Medicine, Tokyo, Japan.
7. LEAP, Japan Agency for Medical Research and Development, Tokyo, Japan.

**Corresponding author:** Masaru Suzuki, M.D., Ph.D.

Department of Respiratory Medicine, Faculty of Medicine and Graduate School of Medicine, Hokkaido University, Sapporo, Japan

North 15 West 7, Kita-ku, Sapporo 060-8638, Japan

E-mail: [suzumasa@med.hokudai.ac.jp](mailto:suzumasa@med.hokudai.ac.jp)

**Supplementary figure legends**

**Figure S1. Gene expressions in the lungs from PPE-treated mice**

Gene expression in the lungs during PPE treatment was assessed using qRT-PCR. (A) IL-1β, (B) IL-5, (C) IL-6, (D) MMP--9, (E) CCL2, (F) TNF-α, (G) IFN-γ.

Data are expressed as mean ± SEM (n = 3-5). ^✻^p <0.05 by the Mann–Whitney U test.


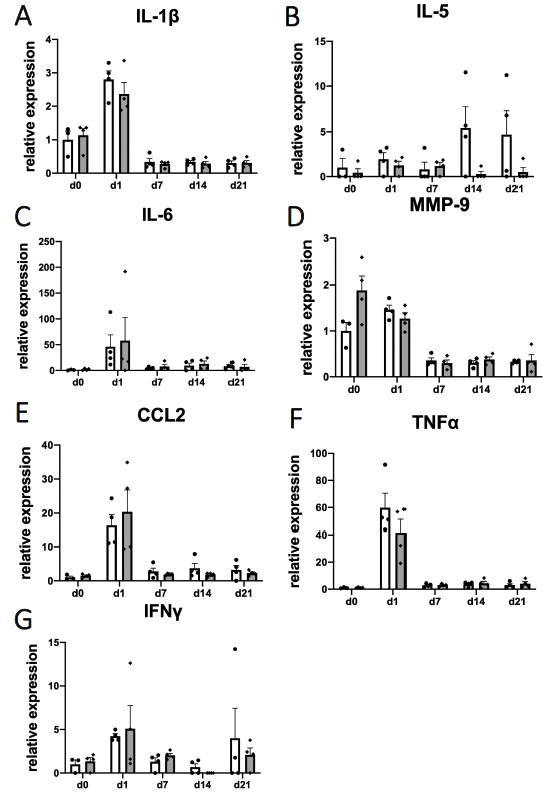


| **Table S1. Characteristics in the study on patients with different smoking status (n=50)** | | | | |
| --- | --- | --- | --- | --- |
|  | **Nonsmokers**  **(n=16)** | **Healthy smokers**  **(n=15)** | **Smokers with**  **COPD**  **(n=19)** | **p value** |
| **Age, years** | 70.7 ± 10.5 | 64.5 ± 8.3 | 65.6 ± 8.7 | 0.21 |
| **Male, n (%)** | 15 (93.8) | 11 (73.3) | 17 (89.5) | 0.22 |
| **Pack-years** | 1.7 ± 3.3 | 45.9 ± 26.3 | 53.8 ± 19.9 | <0.001 |
| **Current smoker, n (%)** | 0 (0) | 7 (43.8) | 9 (47.4) | 0.004 |
| **FEV_1_, % predicted** | 125.2 ± 16.5 | 104.5 ± 17.7 | 65.0 ± 19.5 | <0.001 |
| **FEV_1_/FVC, %** | 77.2 ± 3.7 | 76.7 ± 4.4 | 45.8 ± 11.3 | <0.001 |
| **Notes:** Values are expressed as means ± SD or number (%). Statistical analyses were performed using the Kruskal-Wallis test or the chi-square test. | | | | |
| **Abbreviations**: COPD, chronic obstructive lung disease; FEV_1_, forced expiratory volume in 1 second; FVC, forced vital capacity. | | | | |

| **Table S2. Baseline characteristics in the Hokkaido COPD cohort (n=133)** | | | |  |
| --- | --- | --- | --- | --- |
|  | **Low AIM/IgM (Q1)**  **(n=33)** | **Moderate AIM/IgM (Q2-3)**  **(n=67)** | **High**  **AIM/IgM (Q4)**  **(n=33)** | **p value** |
| **Age, years** | 67.1 ± 9.3 | 70.2 ± 8.2 | 72.6 ± 6.1 | 0.046 |
| **Male, n (%)** | 29 (87.9) | 61 (91.0) | 32 (97.0) | 0.39 |
| **Body mass index, kg/m^2^** | 21.6 ± 3.2 | 22.3 ± 3.4 | 21.8 ± 3.1 | 0.64 |
| **GOLD stage 1 / 2 / 3 / 4** | 12 / 14 / 5 / 2 | 19 / 32 / 15 / 1 | 7 / 12 / 10 / 4 | 0.21 |
| **Pack-years** | 61.9 ± 28.4 | 63.3 ± 32.5 | 62.0 ± 25.9 | 0.96 |
| **Current smoker, n (%)** | 13 (39.4) | 11 (16.4) | 11 (33.3) | 0.03 |
| **FEV_1_, % predicted** | 69.0 ± 25.4 | 66.4 ± 20.4 | 60.5 ± 25.7 | 0.26 |
| **FEV_1_/FVC, %** | 53.2 ± 11.1 | 52.0 ± 12.8 | 48.5 ± 13.1 | 0.37 |
| **Bronchodilator responsiveness**  **of FEV_1_, %^＊^** | 15.2 ± 15.5 | 12.0 ± 11.6 | 12.6 ± 13.5 | 0.90 |
| **DL_CO_, % predicted** | 81.7 ± 22.5 | 78.1 ± 24.0 | 73.5 ± 18.8 | 0.42 |
| **K_CO_, % predicted** | 62.8 ± 19.5 | 67.3 ± 25.0 | 61.9 ± 20.6 | 0.37 |
| **Annual decline in FEV_1_, mL/year**^†^ | -6.2 ± 4.7 | -6.6 ± 4.1 | -6.2± 4.4 | 0.85 |
| **CT emphysema score** | 1.3 ± 1.0 | 1.5 ± 0.9 | 1.7 ± 4.3 | 0.19 |
| **mMRC dyspnea score ≥2, n (%)** | 16 (48.5) | 36 (53.7) | 32 (97.0) | 0.86 |
| **SGRQ total score** | 30.8 ± 15.8 | 29.5 ± 15.1 | 30.2 ± 20.0 | 0.93 |
| **Exacerbation, events/year^‡^** | 0.11 ± 0.29 | 0.12 ± 0.30 | 0.18 ± 0.33 | 0.32 |
| **Notes:** Values are expressed as means ± SD or number (%). Statistical analyses were performed using the Kruskal-Wallis test or the chi-square test.  **^＊^**n=132; ^†^n=124; **^‡^**n=130. | | | | |
| **Abbreviations**: COPD, chronic obstructive lung disease; GOLD, Global Initiative for Chronic Obstructive Lung Disease; FEV_1_, forced expiratory volume in 1 second; FVC, forced vital capacity; DL_CO_, carbon monoxide diffusion capacity; K_CO_, carbon monoxide transfer coefficient; CT, computed tomography; mMRC, modified medical research council; SGRQ, St. George's respiratory questionnaire. | | | | |

| **Table S3. Risk factors for prognosis in the Hokkaido COPD cohort** | | | | | | |
| --- | --- | --- | --- | --- | --- | --- |
|  | **Time to first exacerbation** | | **All-cause**  **mortality** | | **Respiratory-cause mortality** | |
|  | **HR**  **(95% CI)** | **p value** | **HR**  **(95% CI)** | **p value** | **HR**  **(95% CI)** | **p value** |
| **Serum AIM/IgM ratio**  **(1 increase in natural logarithm)** | 2.27  (0.92-5.57) | 0.07 | 2.40  (1.11-5.17) | 0.03 | 5.41  (1.73-16.85) | 0.004 |
| **Age (1 year older)** | 1.03  (0.99-1.07) | 0.17 | 1.11  (1.06-1.16) | <0.001 | 1.13  (1.05-1.21) | 0.001 |
| **Current smoking** | 0.91  (0.46-1.81) | 0.80 | 0.88  (0.61-2.09) | 0.69 | 0.81  (0.27-2.44) | 0.71 |
| **Notes:** Statistical analyses were performed using multivariate Cox proportional hazards model. | | | | | | |
| **Abbreviations**: COPD, chronic obstructive lung disease; HR, hazard ratio; CI, confidence interval. | | | | | | |
